# Blazing the trail for innovative tuberculosis diagnostics

**DOI:** 10.1007/s15010-023-02135-3

**Published:** 2023-11-30

**Authors:** Seda Yerlikaya, Tobias Broger, Chris Isaacs, David Bell, Lydia Holtgrewe, Ankur Gupta-Wright, Payam Nahid, Adithya Cattamanchi, Claudia M. Denkinger

**Affiliations:** 1 Division of Infectious Diseases and Tropical Medicine, Heidelberg University Hospital and Faculty of Medicine, Heidelberg University, Heidelberg, Germany; 2 Connected Diagnostics Limited, London, UK; 3 Independent Consultant, Lake Jackson, TX USA; 4 Institute for Global Health, University College London, London, UK; 5 UCSF Center for Tuberculosis, University of California San Francisco, San Francisco, CA USA; 6 German Centre for Infection Research, Partner Site Heidelberg University Hospital, Heidelberg, Germany; 7 Division of Pulmonary Diseases and Critical Care Medicine, University of California Irvine, Irvine, CA USA

**Keywords:** Diagnostics, Tuberculosis, COVID-19, Innovation, Technology

## Abstract

The COVID-19 pandemic brought diagnostics into the spotlight in an unprecedented way not only for case management but also for population health, surveillance, and monitoring. The industry saw notable levels of investment and accelerated research which sparked a wave of innovation. Simple non-invasive sampling methods such as nasal swabs have become widely used in settings ranging from tertiary hospitals to the community. Self-testing has also been adopted as standard practice using not only conventional lateral flow tests but novel and affordable point-of-care molecular diagnostics. The use of new technologies, including artificial intelligence-based diagnostics, have rapidly expanded in the clinical setting. The capacity for next-generation sequencing and acceptance of digital health has significantly increased. However, 4 years after the pandemic started, the market for SARS-CoV-2 tests is saturated, and developers may benefit from leveraging their innovations for other diseases; tuberculosis (TB) is a worthwhile portfolio expansion for diagnostics developers given the extremely high disease burden, supportive environment from not-for-profit initiatives and governments, and the urgent need to overcome the long-standing dearth of innovation in the TB diagnostics field. In exchange, the current challenges in TB detection may be resolved by adopting enhanced swab-based molecular methods, instrument-based, higher sensitivity antigen detection technologies, and/or artificial intelligence-based digital health technologies developed for COVID-19. The aim of this article is to review how such innovative approaches for COVID-19 diagnosis can be applied to TB to have a comparable impact.

## Introduction

“Squarely put, the drugs are in the north and the disease is in the south,” stated the former World Health Organization (WHO) director general Gro Harlem Brundtland in response to the AIDS epidemic in the late 1990s [1]. The same principle still holds true for innovative tools from drugs to diagnostics today. The COVID-19 pandemic exposed the strengths and weaknesses of the present research and development (R&D) system. While R&D progressed at an unparalleled rate, access to its products outside of the global north and novel technology usage for diseases associated with poverty primarily affecting the global south have remained restricted. One of these diseases, tuberculosis (TB), stands to gain significantly from the diagnostic industry’s surge of innovation brought on by the pandemic. In this review, we explore how the cutting-edge solutions created for COVID-19 diagnosis may be applied to TB to truly impact how TB is diagnosed.

## COVID-19: a new era in diagnostics

### Unprecedented speed and money

In the early stages of the pandemic, WHO established access to the COVID-19 Tools Accelerator (ACT-A) partnership [2] with a budget of $1.5 billion USD (all dollar amounts are given in USD hereafter) [3] for its diagnostic pillar. In the United States (US) alone, the National Institutes of Health (NIH) allocated more than $1.5 billion to the Rapid Acceleration of Diagnostics (RADx^®^) initiative [4–6]. The large investments and sizeable market prospects led to expedited diagnostic research and a surge of innovation. Three months after COVID-19 was declared a pandemic, WHO listed the first two molecular assays for emergency use [7]. As of October 15, 2023, FIND, the global alliance for diagnostics, COVID-19 Test Directory lists 2195 tests including laboratory, point-of-care (POC), and at-home/over-the-counter (OTC) tests, while the Johns Hopkins Centre for Health Security Antigen and Molecular-based Tests Tracker lists 208 commercial and 143 laboratory-developed tests (as of March 30, 2022) [8, 9].

### The best of innovation: new sampling methods and testing technologies

COVID-19 diagnostic test development does not only stand out for its sheer number of tests but also for versatility in terms of targets, sample types, and operational characteristics. Reverse-transcription quantitative polymerase chain reaction (RT–qPCR) on isolated RNA from nasopharyngeal (NP) specimens has been the gold standard for SARS-CoV-2 detection since the beginning of the pandemic; however, the variety of samples and tests expanded quickly in response to the need for effective and practical testing in many contexts and situations. NP swabs were the sample of choice despite sputum having higher average viral load levels because not all patients can produce sputum [10, 11]. Several swab types (e.g., nylon flocked swabs) and swab samples (e.g., NP, oropharyngeal, and nasal swabs) have been evaluated for SARS-CoV-2 detection to optimize sample collection and processing for follow-up molecular or antigen testing [10, 11].

The number and variety of fully-integrated, cartridge-based, rapid molecular platforms that use swabs for sampling has also increased dramatically for COVID-19. As of October 15, 2023, the FIND test tracker includes 34 such commercial tests from 27 companies [8]. The majority of these tests require minimal sample preparation, operate on portable or easily transportable equipment, and provide results during a single clinical visit (within two hours of sample collection) [12]. Reverse transcription loop-mediated isothermal nucleic acid amplification (RT-LAMP) has emerged as the method of choice among the various isothermal techniques due to its low resource requirement and relative simplicity [13]. The main contributing factor to a rise in LAMP-based diagnostics has been the expiration of Eiken Chemical’s key patent on LAMP [29, 30]. The US FDA has authorized the emergency use (EUA) of 18 COVID-19 RT-LAMP-based products by 14 developers (as of October 19, 2023) [14]. Among them are at-home/OTC diagnostic tests and systems utilizing clustered regularly interspaced short palindromic repeat (CRISPR)-Cas (CRISPR-associated protein) for detection after RT-LAMP amplification. As reviewed elsewhere [15–19], CRISPR-Cas shows promise of low-cost, simple, quick, and accurate molecular tests. However, only a small number of CRISPR tests have been approved for use in Clinical Laboratory Improvement Amendments of 1988 (CLIA)-certified laboratories [8, 14], due primarily to its subpar application for POC up to this point.

COVID-19 antigen detection tests have been used for screening and triage throughout the pandemic because they are faster, less expensive than molecular tests, and simple enough to allow for self-testing. Despite their inferior sensitivity compared to PCR, some new POC-applicable instrument-based antigen tests outperformed their conventional lateral flow counterparts [20, 21]. For instance, a systematic review and meta-analysis found the SARS-CoV-2 antigen test from UK-based LumiraDx to be the most sensitive test, with a pooled sensitivity of 82.7% (95% confidence interval [CI] 73.2–89.4%) [20]. Multiple instrument-based, fully automated, POC antigen detection platforms now have US FDA EUA [22] or Conformité Européene (CE)-marking [8].

Breath tests could potentially detect COVID-19 earlier because volatile organic compounds (VOCs) first appear in breath during the early stages of infection [23]; however, only a few breath-based tests have been approved for clinical use [24, 25]. US-based InspectIR Systems obtained US FDA EUA for its Breathalyzer test, which uses a portable gas chromatography-mass spectrometry (GC–MS) instrument to detect five VOCs associated with SARS-CoV-2 infection in exhaled breath [24]. Additionally, the companies Deep Sensing Algorithms (DSA; Finland) and Imspex Diagnostics (UK) received CE-marking for their spectroscopic-based breath tests to determine a person’s metabolic response to COVID-19 [26, 27]; however, their real-world effectiveness and applicability remain to be seen. Moreover, although still in the preclinical stages, exhaled breath aerosol (XBA) collection paired with SARS-CoV-2 molecular detection is showing promise [28–31]. Unlike VOCs, direct nucleic acid or antigen-based pathogen detection in XBA has the potential to be highly specific and is associated with the transmission of respiratory pathogens. Although highly technical and resource-intensive, current tools can effectively collect XBAs and detect pathogens [32]. The COVID-19 pandemic has sparked research into simpler, filter-based XBA collection devices that can be implemented in clinical settings such as face masks with embedded biosensors and blow tubes [28, 33, 34]. Simple blow tubes may offer a more scalable solution with faster sampling. A silicon chip-based sample collector for subsequent molecular detection of SARS-CoV-2 virus particles is being developed for this reason by IMEC, a Belgian R&D organization, in collaboration with industry partners [35, 36].

### Putting diagnostic ownership into patients’ hands: self-testing

The availability of simple, easy-to-use, rapid, and affordable diagnostics, and evidence showing that easy-to-collect samples like saliva and nasal swabs are clinically valid, has made self-testing a viable option. WHO recommended COVID-19 self-testing using rapid antigen tests as a supplemental testing approach in March 2022 [37]. Importantly, for the first time, four molecular test developers received US FDA EUA for self-testing [14]. These tests can be performed with self-collected nasal swabs using portable, battery-powered devices that return results within 20–60 min [8, 38–41]. Although technological and regulatory advancements in this area have been promising, it is still unclear how frequently and effectively molecular home tests are used.

### Next-generation sequencing

Throughout the pandemic, the SARS-CoV-2 genomic surveillance relied heavily on next-generation sequencing (NGS), and when new variants of concern emerged, NGS capabilities swiftly expanded globally [42]. NGS eventually also proved useful for diagnosing the COVID-19 disease, especially when new variants emerged. FDA EUAs for SARS-CoV-2 NGS tests that may be used in CLIA-certified laboratories for diagnostic purposes have so far been granted to two products [14].

### Increased use of digital health technologies: digitization

In the fight against COVID-19, digitally connected diagnostic tests have proven critical for timely case identification and public health surveillance by enabling instant and accurate patient data transmission to health management information systems, real-time monitoring of disease patterns, and assessment of operational needs. As a result, connectivity is now actively considered when developing diagnostics. In addition, the artificial intelligence (AI) community has developed patient triage and diagnosis support software with a focus on a variety of targets, including chest X-ray (CXR) and ultrasound images, cough, and lung sounds [43–49]. However, the small size and low quality of algorithm training data and the difficulty of the regulatory pathways for such solutions have been a barrier for developers, preventing the tools from reaching clinical settings [50]. Nevertheless, the US FDA has granted EUA to three COVID-19 screening devices using machine learning [51]. Moreover, a smartphone-based cough sound app for COVID-19 screening (ResApp Health, AU) with a CE-mark and approval from the Australian Therapeutic Goods Administration (TGA) now establishes a precedent for the regulatory approval of AI-based tools for infectious disease detection [52, 53].

## What about TB?

TB still claims 1.5 million lives every year, despite COVID-19 having displaced it as the world’s most lethal infectious disease [54]. Delayed and absent diagnoses provide a substantial barrier to the improvement of individual TB outcomes and control. Each year, more than one-third of all cases of TB go undiagnosed. This diagnostic gap has widened due to COVID-19 [54]. Sputum smear microscopy is still the most widely used TB microbiological test, even though WHO recommends rapid molecular testing first [55]. The varying clinical performance of smear microscopy, together with the difficulties in collecting sputum from patients and access to healthcare, is one of the primary reasons for missed TB diagnosis.

**Fig. 1:**
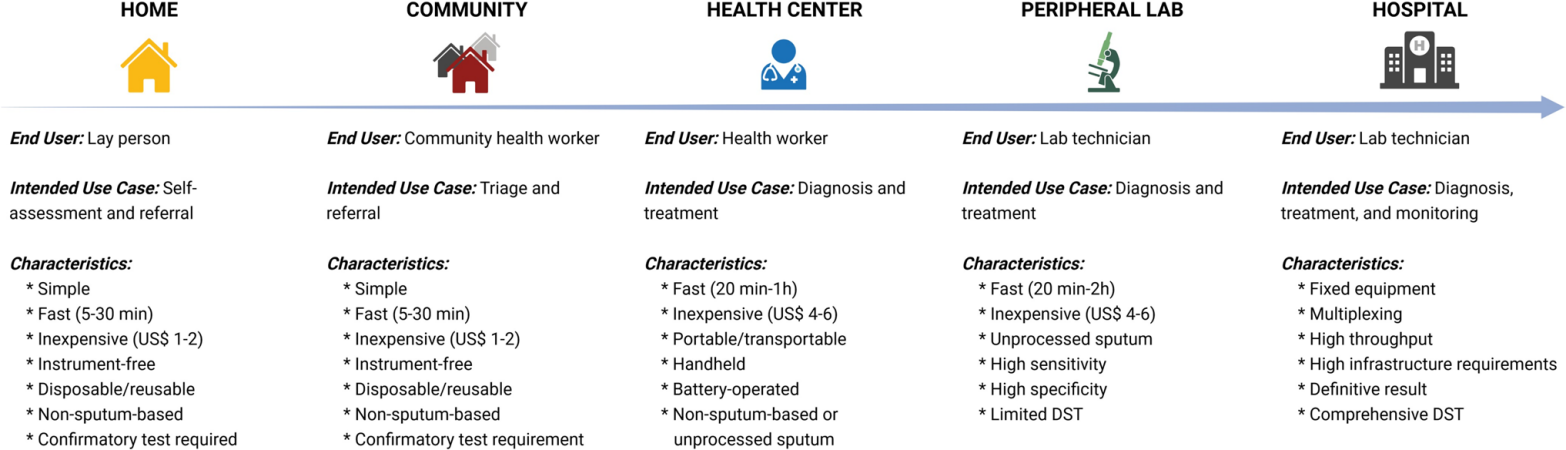
A summary of TB diagnostic needs along the care cascade (adapted from 160 with author permission). *DST* drug-susceptibility testing. Figure created with BioRender.com

To foster innovation in TB diagnostics and to link end-user demands with test targets and specifications, WHO released high-priority target product profiles (TPPs) for novel TB diagnostics in 2014 [56]. In order to address the local demands at all levels of healthcare in high-TB settings, a variety of technologies in the field of TB diagnostics are required, as reflected by the WHO TPPs (Fig. 1). Despite this, no TB test has satisfied these targets. Accelerated innovation is critically needed to ensure that novel, effective, and fit-for-purpose diagnostics reach the market and aid in the search for the “missing millions” [54, 57].

### Money and investment

UN member states recognized the need for an annual TB budget of US$ 2 billion in 2018 [58]; nonetheless, funding for TB research has not increased since then [59]. Moreover, the amount of money spent globally on TB research remained at US$ 915 million in 2020, falling short of even half of the UN target or the total budget for the ACT-A diagnostics pillar [54]. In reality, it is estimated that in the next 2 years, about US$10 billion will be required, US$ 613 million of which will go towards diagnostics research [59].

Globally, an estimated 10.6 million people fell sick with TB in 2021, nearly 90% live in 30 high TB burden countries, all of which are LMICs [54]. An analysis of the smear-replacement market from 2014 pegged the market's size at 30.8 million tests, with a potential annual market value of US$ 154 million, assuming a US$ 5-unit cost [60]. This estimate only considers the initial diagnosis; non-sputum, biomarker-based tests, screening or drug susceptibility testing are not included. In addition, Kik et al. conducted a thorough analysis to estimate the market potential of a non-sputum-based biomarker test for the high-burden countries of South Africa, Brazil, China, and India, and they estimated a potential value of US$ 56–84 million and 14 million tests for these four countries [61–64]. The market for non-sputum, biomarker-based tests is predicted to reach US$ 406 million by 2026, growing at a compound annual growth rate (CAGR) of about 6%, applying the same assumptions across all LMICs. Moreover, in high-burden countries, TB diagnostics are purchased for the public healthcare sector through national health ministries, which are financially backed by global health donors; however, there is also a sizable private healthcare sector that requires TB diagnostics, aside from the heavily involved public sector.

Despite this market potential, companies steer clear of the TB diagnostics sector, fearing substantial opportunity costs associated with prioritizing products for the LMIC market. Nonetheless, it is worthwhile to consider that any developer is likely to benefit from economies of scale by producing low-margin, but high-volume products and expanding its portfolio, as Cepheid did in the case of GeneXpert Dx System (Cepheid, CA, USA) GeneXpert System. Besides, some of the countries with high TB burden are emerging markets [65]. Moreover, several clinical platforming initiatives offer developers in-kind contributions, reducing development costs and accelerating time to market. Through its ‘Feasibility of Novel Diagnostics for TB in Endemic Countries (FEND for TB)’ program, the NIH is currently funding three such initiatives; ENDxTB (http://www.endxtb.com), R2D2 TB Network (http://www.r2d2tbnetwork.org), and FEND-TB (http://www.fend-tb.org) [66]. Additionally, global organizations like FIND and Stop TB Partnership provide developers interested in neglected, poverty-related diseases with ongoing support and guidance for development, validation, and scale-up [67, 68]. These resources provide diagnostic developers, including academic groups, start-ups, and companies, with access to clinical samples and clinical evaluations for TB diagnostic technologies at any stage of development. Such initiatives are likely to continue. For instance, the Supporting, Mobilizing, and Accelerating Research for Tuberculosis Elimination (SMART4TB) project, recently funded by the US Agency for International Development (USAID), intends to support the next step toward large-scale implementation of innovative tools [69].

To ensure a sustainable supply of diagnostics in settings of need, it is crucial to leverage such initiatives to also advance local R&D and manufacturing in LMICs, as demonstrated for COVID-19 with the IPD/DiaTROpix project in Senegal [70–72]. Increased collaboration between LMIC-based diagnostic developers and clinical platform networks like those mentioned above, as well as with international groups like FIND and the Stop TB Partnership, is likely to give them the support they need to enter the global diagnostics scene and eventually reduce reliance on high-income country-based companies for TB tests.

### Challenge: the bug

The biology of SARS-CoV-2 permitted the development of rapid, POC diagnostics, but the adaptation to TB presents technical challenges. The lipid-rich cell wall of the *Mycobacterium tuberculosis* (MTB) bacterium makes chemical and enzymatic lysis methods mostly ineffective, often necessitating mechanical lysis methods like bead-beating and sonication instead. The yield and quality of genomic DNA are consequently frequently adversely affected. Further reducing the amount of MTB genomic DNA available for subsequent molecular detection is the low bacterial load in accessible clinical samples (e.g., oral swabs). The design of PCR assays is further complicated by the high guanine/cytosine content of the MTB genome. Because of these issues, the methodologies for sample selection, bacterial lysis, nucleic acid extraction, and PCR design must be carefully considered when working with MTB.

Compared to well-defined, easily detectable viral antigens, few antigenic biomarkers have been identified for MTB, with lipoglycan lipoarabinomannan (LAM) being the most researched, most promising and conveniently accessible from an easy-to-collect sample, urine [73]. LAM is easier to detect with acceptable performance in individuals with advanced HIV in those with disseminated TB [74]. LAM, however, shows structural variations among MTB complex species and different bodily fluids [75, 76]. Additionally, current research suggests that cultured LAM is not entirely representative of LAM in patients [77], making the use of purified urinary LAM crucial for R&D.

### Technical innovation: sampling

**Fig. 2:**
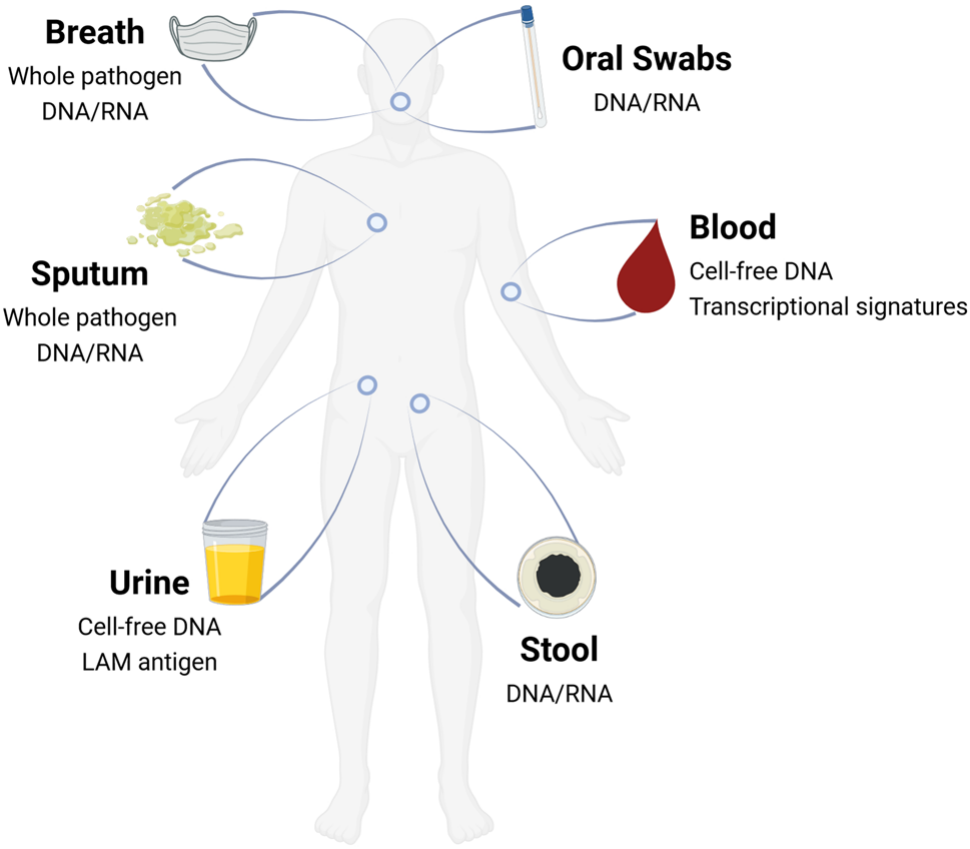
Clinical samples and targets proposed for TB testing. Figure created with BioRender.com

Sample type is an important consideration when applying novel technologies to TB. Sputum is the sample of choice for TB, but it is difficult to work with and challenging to collect, especially from children and people living with HIV. As a result, the R&D focuses on moving away from sputum and towards more accessible and easy-to-collect samples for TB diagnosis [56] (Fig. 2).

Rapid, POC molecular platforms compatible with swab samples have been extremely useful for COVID-19 testing. Oral swabs have emerged as an appealing sample choice for TB testing as well. The feasibility of employing oral swabs for TB testing has already been documented in the literature [78–91]. The sensitivity of Xpert MTB/RIF Ultra (Xpert Ultra; Cepheid, CA, USA) when used with oral swabs ranged from 45% (95% CI 29–62%) to 77.8% (95% CI 64.4–88.0%) compared to a sensitivity of ~ 90% for sputum [80, 83, 92]. The performance discrepancies can largely be explained by the diverse swabbing and sample-handling strategies employed. For instance, the Cangelosi and Franke groups showed that tongue swabs yield stronger signals than cheek or gum swabs and that various swab brands can differ by up to two-fold in the bacterial mass they can capture [79, 85, 87]. This suggests that performance is likely to increase with swab type, collection, and storage optimization. In line with this, multiple concurrent efforts from several groups are underway to optimize and standardize the collection and handling of oral swabs for TB testing. Once an optimal sampling protocol is devised, swabs are expected to make it easier to adapt a COVID-19 test strategy to TB. Even if the sensitivity does not reach that of sputum, swab-based TB tests may reach high diagnostic yields through increased sample availability and be helpful when sputum cannot be obtained for molecular testing. Moreover, swabs collected in communities could be used for high-throughput testing with WHO-approved moderate-complexity molecular technologies, which had more installed bases during the pandemic, if the sample referral network was improved. This would facilitate the annual community-wide TB screenings, which have been shown to lower the prevalence [93]. Additionally, COVID-19 has also helped to improve swab sampling’s acceptance and perception. The use of swabs for sample collection for diagnostic purposes is now commonplace among populations worldwide. This could be leveraged to increase swab uptake and acceptability as a sample for TB diagnosis.

Breath has long been an attractive diagnostic sample for TB due to its non-invasive collection and link to TB transmission. VOC-based detection in exhaled breath and condensate, which is sensitive to fluctuations in exogenous and endogenous variables, has long been the focus of breath-based testing for TB [23, 94–96]. While other VOC-based breath tests had a wide range of sensitivity (62% to 100%) and specificity (11% to 84%), electronic nose tests were reported to have an estimated summary sensitivity and specificity of 92% (95% CI 82–97%) and 93% (95% CI 88–96%), respectively [97]; however, clinical use of breath-based TB testing has not yet reached its full potential. The main barrier to its introduction into clinical settings and broad use has been the lack of standardization of breath samples and analysis, which results in high test performance variability [95]. The development and application of VOC-detecting breath tests for TB may be sped up by the innovative methodologies and detecting techniques that have just hit the market during the COVID-19 period. Alternatively, XBA bears the promise of being a sensitive and highly specific diagnostic specimen due to its capacity to carry pathogens, thereby enabling pathogen detection via molecular tests [95, 98]. The key challenge to detect pathogen nucleic acids in XBA is their low abundance. Efficient aerosol collection for MTB detection has up until now required intensive technical efforts or lengthy sampling periods and has only been used in academic research. The respiratory aerosol sampling chamber (RASC), a 1.4 m^3^ cleanroom chamber where patients sit for XBA sampling that requires active pumping of large volumes of air, serves as an illustration of this [99]. Although TB was detected in 97% of patients after 10 min of sampling, such complex instrumentation is unsuitable for low-cost POC use. Face mask sampling and filter-bearing blow tubes, which have been suggested for COVID-19 detection, could be further developed to detect TB as more POC solutions, particularly in light of the currently available proof-of-concept data for the applicability of face mask sampling to TB [100–104].

The suitability of other sample types for TB testing (e.g., urine, blood, stool) has also been assessed. Since LAM is found in TB patients’ urine, urine has been the sample of choice for antigen detection tests [105]. Although the use of urine as a sample type for molecular TB testing has also been investigated, the results show that it is less accurate than sputum-based testing, with a pooled sensitivity of 55% (95% CI 36–72%) [106]. Host blood transcriptomic TB biomarkers for diagnostic, prognostic, and treatment monitoring purposes are also being investigated [107–109]. Despite this, they are still a long way from being implemented in clinical settings due to their variable performance, as well as the high cost and complexity of the currently available detection tools [108]. Cell-free DNA (cfDNA) is another biomarker possibly found in urine and blood (cfDNA). Currently, the pooled sensitivity of cfDNA for TB diagnosis using various sample types is reported to be 68% (95% CI 52–80%) [110]; however, with optimized pre-analytical conditions and more sensitive detection methods, as seen in COVID-19, this is anticipated to improve [111–113]. Additionally, proof-of-concept for the detection of MTB cfDNA using CRISPR in both adults and children with TB has been achieved, opening the door for the use of future CRISPR-based POC solutions for TB [114]. Given the challenges in collecting sputum samples from children, WHO advised utilizing Xpert MTB/RIF (Xpert; Cepheid, CA, USA) testing of stool samples as a primary diagnostic test for TB in children presenting pulmonary TB signs and symptoms in 2020 [115]. Its application in adults has also been researched, and the pooled sensitivity of stool PCR was reported to be 89.7% (95% CI 81.4–95.9%) [116].

### Technical innovation: instrumentation

#### Molecular testing

Since its introduction in 2010, the GeneXpert Dx System (Cepheid, CA, USA), an integrated, single-use cartridge-based diagnostic system, has been the molecular diagnostic test of preference for TB [117]. MTB DNA and mutations linked to rifampicin resistance are detected by the Xpert and Xpert Ultra cartridges, the latter of which is an upgraded model with greater sensitivity [117, 118]. The current global access price of the GeneXpert 10-color module is less than US$ 10,000, while the Xpert and Xpert Ultra cartridges cost about US$ 7.97 apiece, according to the Stop TB Partnership Global Drug Facility (GDF) Diagnostics, Medical Devices and Other Health Products Catalog [119]. Xpert is, nonetheless, not a true POC solution because it requires constant power, high maintenance due to high susceptibility to dust, and low operating temperatures [120]. GeneXpert Omni System (Omni), a sample-to-result molecular diagnostics system that can run all of the company’s molecular diagnostic test cartridges, was introduced by Cepheid in 2015 as a genuine POC substitute. It is easy to use, portable, battery-operated, and smartphone-controlled [121]. Despite Omni’s demonstrated clinical performance in independent studies [121], Cepheid recently halted Omni’s development following several delays [122]. This leaves a sizable market for innovative, near-patient molecular POC solutions for TB testing.

The Truenat^™^ System (Molbio Diagnostics, India) is now the sole POC option for TB. Truelab^™^ is a chip-based micro-PCR device that can perform a 40-cycle real-time PCR in 35 min, while Trueprep^®^ is an automated device that extracts DNA from sputum in under 20 min. Both are portable, battery-operated, and robust even in harsh environmental conditions (i.e., 40 ℃, 80% relative humidity) [123, 124]. The Truenat^™^ MTB and MTB Plus assays for TB detection and the MTB-RIF Dx reflex assay for RIF resistance detection are three assays that may be run on Truelab^™^ utilizing the DNA eluate from Trueprep^®^. WHO recommended Truenat as an initial test for TB and rifampicin resistance detection after independent clinical trials showed that the assays have comparable accuracy to Xpert and Xpert Ultra, even when used in primary health care clinics [55]. The Stop TB Partnership supports the introduction of Truenat^™^ with 301 devices and 580,000 tests in countries with a high TB burden [125]. Truelab^™^ Uno Dx Workstation is listed as costing US$ 10,000 in the Stop TB Partnership GDF catalog, whereas single MTB and MTB Plus tests are listed as costing US$ 7.9 apiece [119].

Less than 40% of all notified TB patients undergo an initial rapid diagnostic test, creating a significant diagnostic gap [126]. The TB field would benefit immensely from a variety of POC solutions employable in community or at-home settings, notwithstanding the promise of Truenat^™^ as a near-patient TB test. For more accessible TB diagnosis, instrument-free molecular tests compatible with such use cases are highly desirable. Once the oral swab procedures for TB testing are refined and made widely applicable by diagnostic developers, the swab-based nature of these tests makes their application to TB easier. Nevertheless, TB-related technical challenges persist. The preferred site for TB swab collection, the dorsum of the tongue, appears to have a lower bacterial load than sputum. Consequently, highly sensitive systems are needed. CRISPR could enable such higher sensitivities when combined with isothermal methods, but its POC application would require one-pot techniques and automation [127].

The COVID-19 POC tests’ current price, nonetheless, is a deterrent to their adoption in LMICs; however, large-scale manufacturing spurred by promising market opportunities and bulk purchases for LMIC markets combined with a tiered pricing approach is likely to drive the test prices down, at least for high-burden settings in need. Additionally, the current WHO recommendation for the adoption of LAMP-based tests for TB would make it easier for any future LAMP tests to be prequalified and get access to the market [55]. Besides, the expansion of COVID-19 molecular platforms installed globally would facilitate the quick adoption of the newly developed TB assays for these platforms. In addition, using open source tools, such as the no-cost licenses provided by UC Berkeley and Lawrence Livermore National Lab for the best primer sets for a TB LAMP assay that they identified using a comparative genomics method, can also help to ease the adoption of LAMP-based novel testing for TB (unpublished data).

#### Antigen testing

The first urinary antigen detection test for TB was the Alere Determine^™^ TB LAM Ag test (Abbott, IL, USA), which was recommended by WHO for TB diagnosis and screening in people living with HIV [128, 129]. The test was an intriguing alternative for usage in resource-limited situations, because of its quick turnaround time (< 30 min), instrument-free operation, and minimal training requirements with its global access price at US$ 3.70 per test [119, 130]. It was also the only commercially available non-sputum-based TB test since it detected LAM in urine; however, its adoption, even in settings with a high HIV/TB burden, remained limited due to its suboptimal performance [131]. In an effort to enhance analytical and clinical performance, Fujifilm (JP), with support and guidance from FIND, developed a new LFA called Fujifilm SILVAMP TB LAM that also detects LAM in urine and returns results in under an hour [132]. The test performance of this next-generation of LAM-based urine tests approaches the WHO TPP for a non-sputum-based, biomarker test (≥65% sensitivity; ≥98% specificity) [56, 133]. Improved performance of the test as compared to its Alere equivalent was made possible by the employment of higher affinity monoclonal antibodies and a silver-amplification phase that boosts the visibility of the test and control lines [134, 135]. The limit of detection (LoD) of a rapid, affordable POC LAM detection test that can detect TB in all patient groups and meet the WHO TPP is projected to be 5 pg/mL in comparison to the current tests’ LoD of > 25 pg/mL [136]. Instrument-based, high-sensitivity antigen detection approaches, such as those utilized for COVID-19, are therefore more likely to hit this target than conventional LFAs; therefore, fully automated, instrument-based POC antigen detection tools developed for COVID-19 would be worth exploring for their performance in detecting LAM in TB patients. Moreover, well-characterized monoclonal antibodies [76, 134, 137], well-described LAM concentration ranges [136], and readily available and easily accessible biobanks [138, 139] can all be advantageous for the development of next-generation LAM tests with improved performance.

#### Sequencing

The right choice of TB treatment, where drug resistance is a serious issue and a factor in high morbidity and mortality, depends on the detection of clinically relevant drug-resistance genes along with the disease. Molecular and conventional culture-dependent phenotypic methods (i.e., the BD BACTEC^™^ MGIT^™^ 960 system) are currently used in drug-susceptibility testing (DST) for TB [55]; however, culture methods are labor- and time-intensive, and molecular methods can only target a limited number of genes and mutations. WHO reported a total of 196 individual clinically relevant mutations linked to resistance to 12 TB drugs in its 2021 Catalogue of mutations in MTB complex and their association with drug resistance [140]. NGS technology is anticipated to aid in addressing these challenges by enabling comprehensive, rapid DST [141]; however, the use of NGS for TB has so far been constrained by its high cost. It can, nonetheless, be envisaged that NGS-based DST for TB will prove to be cost-effective when and if it can be implemented into the current COVID-19 workflows. Additionally, the application of NGS for TB is projected to be facilitated by the COVID-19-driven expansion of global sequencing capacity, availability of qualified staff, experience in using NGS for clinical purposes, and readily-available data analysis and storage solutions [142]. It is, though, necessary to develop NGS solutions that can be applied straight from sputum (or any other clinical sample) or that are simple to plug into newly developed molecular TB diagnostics.

#### Digital health technologies

The use of digital technology in the field of TB diagnostics has thus far been restricted to the use of computer-aided detection (CAD) software for automatically reading and interpreting chest X-ray (CXR) images, obviating the need for expert readers (radiologists) [143, 144]. COVID-19-driven advances in digital innovation and data science have created new opportunities for better TB care. For instance, the COVID-19 pandemic has accelerated healthcare workers’ digital transformation [145, 146], which can and should be extended to the TB field. Despite evidence of improved data quality and patient management when using digital records and tools, adoption of digital health technologies aimed at healthcare workers has been slow in the TB field [147], in part due to a lack of focus on making them acceptable to intended users and feasible to implement and sustain in low-resource, high-burden settings. The COVID-19 experience, however, is likely to facilitate transitioning to digital, case-based, real-time surveillance systems for TB as advocated by WHO [148]. Therefore, in the post-COVID-19 era, the need for novel TB diagnostic tests with connectivity features is greater than ever.

Building off of the COVID-19 experience, access-related barriers to TB diagnosis may also be addressed by AI-based digital solutions. In fact, several tools to detect and classify cough and lung sounds are being developed now, for applications like TB screening and triage [149–151]. Yet, despite the promise of machine learning algorithms to support clinical decision-making and improve healthcare delivery [152], there are currently no digital tools for TB to help healthcare workers in low-resource settings with patient management that use AI-based prediction models to provide a personalized clinical recommendation based on a TB risk assessment.

The demand for data, however, threatened to further fragment an already complex information landscape with multiple actors and overlapping activities including those who needed to analyze available data and those who wanted to commercially exploit it. There remain many challenges that hamper the effective sharing, analysis and use of data for country decision-making, including the lack of any prevailing business model for sustainability. While the development and evolution of standards applicable to DHTs continue, gaps still persist in the ecosystem, such as infrastructure support and human capacity to introduce, scale up, and sustain these tools. Only if these efforts continue will the diagnostic platforms that were developed during the pandemic and included connectivity be useful for TB.

## Conclusion

Early in the pandemic, the COVID-19 diagnostic market was flooded with technologies because of lucrative funding possibilities and alluring market prospects; however, the market is now saturated. The decision of an increasing number of companies to reduce workforces and close facilities is a reflection of a drop in the demand for COVID-19 testing [153, 154]. The developers, funders, and most crucially the patients in need would all benefit more if the existing potential was directed on diseases like TB where there is a significant need for novel diagnostic tools.

**Fig. 3:**
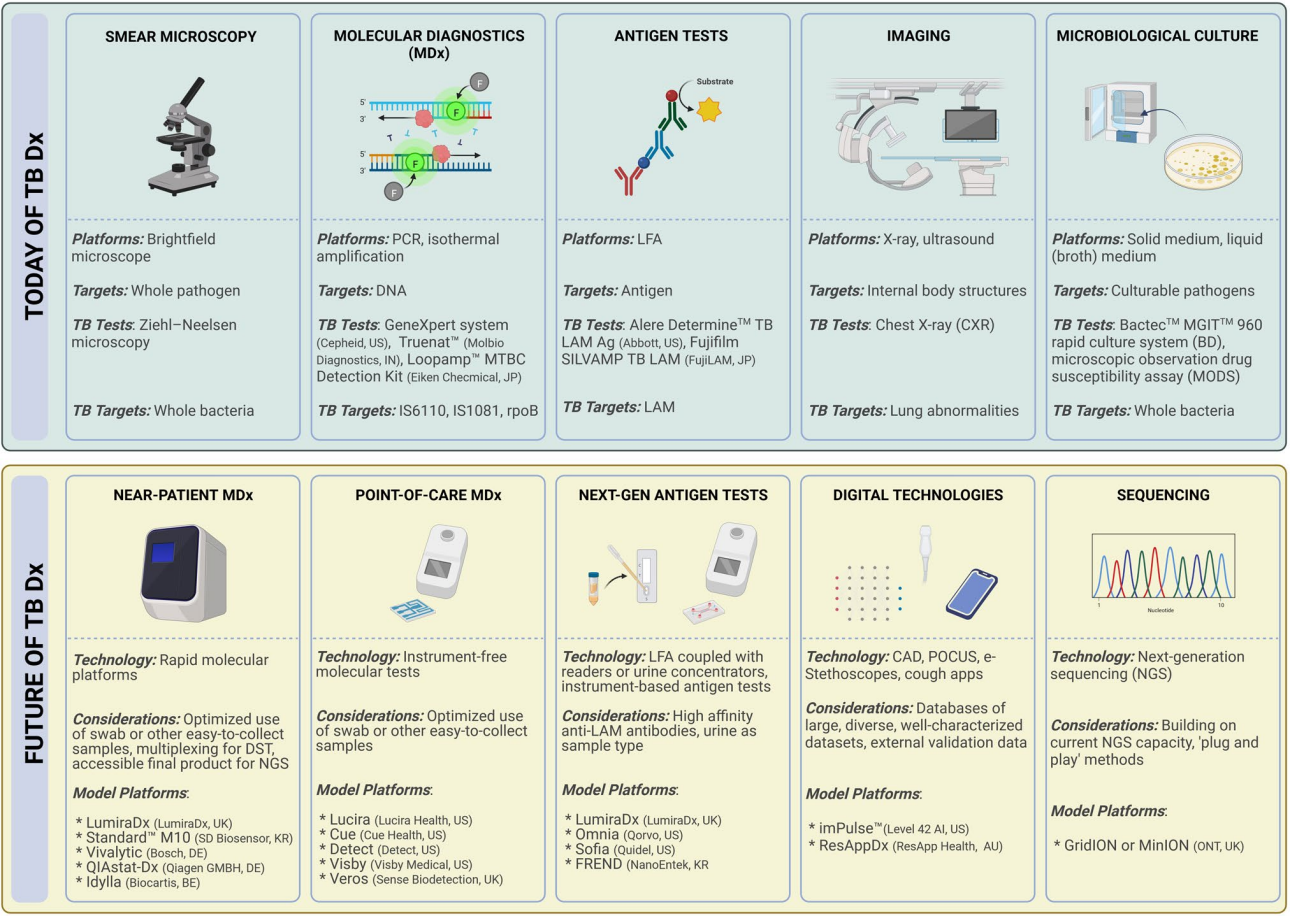
Today and future of TB diagnostics (Dx). IS6110: insertion sequence 6110; IS1081: insertion sequence 1081; *DST* drug-susceptibility testing, *NGS* next-generation sequencing, *LFA* lateral flow assay, *LAM* lipoarabinomannan, *CAD* computer-aided detection, *POCUS* point-of-care ultrasound. Figure created with BioRender.com

The search for a “key” that unlocks all the doors to the diagnostic conundrum of TB has long been a focus in this field (155, 156). The long-sought solution was initially believed to be Xpert, then Omni; however, the failure of such bets on individual technologies to address all needs for controlling or preventing the complex medical and socioeconomic challenges caused by TB should be clear by this point. Instead, a variety of diagnostic possibilities embedded in clinical algorithms are necessary in order to meet local needs in high TB burden settings. With the breadth and variety of technological innovation sparked by the pandemic, COVID-19 presents a unique opportunity in this regard (Fig. 3). Political will and similar investments are required to encourage developers’ interest in the LMIC sector, which is the primary market for TB diagnostics. Long-term success in the TB diagnostics field will also depend on funding local R&D and production and knowledge transfer to LMIC to guarantee a sustainable supply of TB diagnostics.
